# Profiling of Copy Number Alterations Using Low-Coverage Whole-Genome Sequencing Informs Differential Diagnosis and Prognosis in Primary Cutaneous Follicle Center Lymphoma

**DOI:** 10.1016/j.modpat.2024.100465

**Published:** 2024-05

**Authors:** Bence Bátai, Laura Kiss, Luca Varga, Ákos Nagy, Jacob Househam, Ann-Marie Baker, Tamás László, Anna Udvari, Róbert Horváth, Tibor Nagy, Judit Csomor, József Szakonyi, Tamás Schneider, Trevor A. Graham, Donát Alpár, Jude Fitzgibbon, Ágota Szepesi, Csaba Bödör

**Affiliations:** a HCEMM-SU Molecular Oncohematology Research Group, Department of Pathology and Experimental Cancer Research, Semmelweis University, Budapest, Hungary; b Department of Internal Medicine and Hematology, Semmelweis University, Budapest, Hungary; c Genomics and Evolutionary Dynamics Team, Centre for Evolution and Cancer, The Institute for Cancer Research, London, United Kingdom; d Department of Biochemistry and Molecular Biology, Faculty of Medicine, University of Debrecen, Debrecen, Hungary; e Department of Pathology and Experimental Cancer Research, Semmelweis University, Budapest, Hungary; f Department of Dermatology, Venereology and Dermatooncology, Semmelweis University, Budapest, Hungary; g Department of Hematology and Lymphoma, National Institute of Oncology, Budapest, Hungary; h Barts Cancer Institute, Queen Mary University of London, London, United Kingdom

**Keywords:** copy number profiling, cutaneous lymphoma, genomics, next-generation sequencing

## Abstract

Primary cutaneous follicle center lymphoma (PCFCL) has an excellent prognosis using local treatment, whereas nodal follicular lymphoma (nFL), occasionally presenting with cutaneous spread, often requires systemic therapy. Distinction of the 2 diseases based on histopathology alone might be challenging. Copy number alterations (CNAs) have scarcely been explored on a genome-wide scale in PCFCL; however, they might serve as potential biomarkers during differential diagnosis and risk stratification. Low-coverage whole-genome sequencing is a robust, high-throughput method for genome-wide copy number profiling. In this study, we analyzed 28 PCFCL samples from 20 patients and compared the copy number profiles with a cohort of diagnostic samples of 64 nFL patients. Although the copy number profile of PCFCL was similar to that of nFL, PCFCL lacked amplifications of 18q, with the frequency peaking at 18q21.33 in nFL cases involving the *BCL2* locus (PCFCL: 5.0% vs nFL: 31.3%, *P* = .018, Fisher exact test). Development of distant cutaneous spread was significantly associated with higher genomic instability including the proportion of genome altered (0.02 vs 0.13, *P* = .033) and number of CNAs (2 vs 9 *P* = .017), as well as the enrichment of 2p22.2-p15 amplification involving *REL* and *XPO1* (6.3% vs 60.0%, *P* = .005), 3q23-q24 amplification (0.0% vs 50.0%, *P* = .004), 6q16.1-q23.3 deletion (6.3% vs 50.0%, *P* = .018), and 9p21.3 deletion covering *CDKN2A* and *CDKN2B* loci (0.0% vs 40.0%, *P* = .014, all Fisher exact test) in PCFCL. Analysis of sequential tumor samples in 2 cases harboring an unfavorable clinical course pointed to the acquisition of 2p amplification in the earliest common progenitor underlining its pivotal role in malignant transformation. By performing genome-wide copy number profiling on the largest patient cohort to date, we identified distinctive CNA alterations conceivably facilitating the differential diagnosis of PCFCL and secondary cutaneous involvement of nFL and potentially aiding the risk stratification of patients with PCFCL in the future.

## Introduction

Classical or nodal follicular lymphoma (nFL) usually presents as a systemic disease involving the lymph nodes, spleen, and bone marrow, often harboring the *IGH::BCL2* translocation. In contrast, special forms of FL are localized to 1 anatomical region, exhibiting specific clinicopathological features and less frequently harboring the *IGH::BCL2* translocation, hence representing separate disease entities in recent classifications, including primary cutaneous follicle center lymphoma (PCFCL).^1^, ^2^, ^3^ Studies investigating the genetic background of PCFCL revealed common pathways of lymphomagenesis with recurrently mutated genes, including *TNFRSF14*, *CREBBP*, *TNFAIP3*, *KMT2D*, and *EZH2* mutations in both PCFCL and nFL. However, differences in the frequency of recurrent genetic alterations point to specific determinants of disease pathogenesis and can have an important role in differential diagnosis as well as risk stratification.^4^, ^5^, ^6^ Previous genetic studies in PCFCL focused on the investigation of *BCL2* rearrangement status, 1p36 deletion, and targeted sequencing, leaving the genome-wide copy number profile of PCFCL largely unexplored.

Low-coverage whole-genome sequencing (lcWGS) is an emerging molecular diagnostic method, enabling high-throughput, robust identification of genome-wide copy number alterations (CNAs) based on the relative depth of coverage.^7^ This method, utilizing the mapping of single-end sequencing reads to the reference genome and counting those in predetermined genomic windows (called “bins”), is highly efficient at detecting CNAs from fragmented DNA, including circulating cell-free DNA or DNA isolated from formalin-fixed paraffin-embedded tissue samples. Due its cost-effectiveness and high tolerance for input DNA quality, lcWGS is a molecular method with high translational potential. To date, application of the technique in lymphomas has been limited to diffuse large B-cell lymphoma (DLBCL), improving the risk prediction for the patients before chimeric antigen receptor T-cell therapy.^8^

Here, we performed time-resolved lcWGS in a cohort of PCFCL patients to define the complex CNA profile of PCFCL throughout the disease course. In order to identify differences potentially underlying the clinical behavior and having a potential value in differential diagnosis, we compared the observed frequencies in PCFCL to data generated using the same workflow in a cohort of nFL samples. We also investigated temporal changes in PCFCL using serial samples collected during the disease course from 6 patients to explore evolutionary trajectories in this rare, indolent disease.

## Materials and Methods

### Patient Samples

Twenty-eight tissue samples were collected and re-evaluated from 20 PCFCL patients diagnosed between 2001 and 2022 at Semmelweis University, Budapest with available representative tumor tissue at diagnosis and/or recurrence. Clinical data were retrieved from the clinical database and reviewed by the care-provider dermatologist. Clinical examinations included thorough physical and routine laboratory examinations (complete blood cell count and serum chemistry studies), chest X-ray, ultrasonography, and positron emission tomography–computed tomography scan. Peripheral blood and occasionally bone marrow involvement was determined by flow cytometry and histological examination. Affected body regions were identified, and staging was performed based on the International Society for Cutaneous Lymphomas European Organisation for Research and Treatment of Cancer (EORTC) staging guideline for nonmycosis fungoides/Sezary syndrome cutaneous lymphomas.^9^ nFL samples were collected from 64 patients diagnosed between 2002 and 2019 and re-evaluated at Semmelweis University according to the fourth edition of World Health Organization criteria.^10^

The study was conducted in accordance with the Declaration of Helsinki and approved by the Ethics Committee of the Hungarian Medical Research Council (45371-2/2016/EKU and IV/5495-3/2021/EKU). All patients gave informed consent for the research use of archival tissue material and clinical data.

### Histology and Immunohistochemistry

Histological re-evaluation was performed by 2 expert hematopathologists (J.Cs. and A.Sz.) according to the revised World Health Organization and EORTC classification.^11^ The main histological differential diagnosis of PCFCL is reactive follicular hyperplasia, primary cutaneous marginal zone lymphoma, and DLBCL leg type. The histopathological findings differentiating PCFCL from reactive lymphoid proliferations were dense, deep dermal, or subcutaneous infiltration of CD20-positive B cells, clonal expression of IgG light chains, or clonal *IGH* rearrangement; CD10 and/or BCL6 expressions for the vast majority of the tumor cells together with centrocytic cytology excluded primary cutaneous marginal zone lymphoma, whereas nodularity with expanded presence of follicular dendritic cell network, the presence of centrocytes, and MUM1 negativity were the main criteria to exclude leg-type DLBCL.

Immunohistochemical staining was performed on a LEICA Bond Max (Leica Biosystems) system. For antigen localization, DAB polymer (DAKO) was used. Antibodies used for immunohistochemical staining were as follows: CD3 (clone QBEnd10), CD20 (clone L-26), Ki67 (clone MIB-1), BCL2 (clone 124), BCL6 (clone PG.B6p), MUM1 (clone M7259), CD21 (clone 2G9), and CD10 (clone CM139C). Healthy tonsil samples served as negative controls.

The cutoff value for BCL2 positivity was set to 80%, whereas cutoff values for CD10, BCL6, and MUM1 positivity were determined as 30%.

### Interphase Fluorescence In Situ Hybridization

Fluorescence in situ hybridization (FISH) was performed as part of the routine diagnostic workup as described before.^5^

### DNA Isolation

Formalin-fixed, paraffin-embedded tissue samples were sectioned for DNA isolation and the microscopical assessment of tumor cell ratio. In case of low tumor cell purity, tissue blocks were macrodissected to reach a tumor cell content of >50%. DNA was isolated using the QIAamp DNA FFPE Tissue Kit (Qiagen) and quantified using the Qubit HS dsDNA Kit (Thermo Fisher Scientific).

### IGH Clonality Analysis

*IGH* gene rearrangement analysis was carried out for clonality assessment using the BIOMED-2 Concerted Action protocol as part of the routine diagnostic workup using the *IGH* V_H_-J_H_ FR1, FR2, and FR3 primer sets.^12^ The EuroClonality/BIOMED-2 recommendations were followed in the interpretation of the results.^13^

### Sequencing Library Preparation and Low-Coverage Whole-Genome Sequencing

Isolated DNA integrity was determined using a multiplex GAPDH PCR approach outlined by van Beers et al^14^ using 2 ng of DNA input, 12.5 μL of AmpliTaq Gold DNA Polymerase (Thermo Fisher Scientific), and 10 pmol of each primer (Integrated DNA Technologies) in a total reaction volume of 25 μL. PCR products were analyzed on a 2.6% agarose gel with GelRed Nucleic Acid Gel Stain (Biotium). During library preparation, samples with low integrity (≤2 amplification lines) were amplified with 2 additional PCR cycles compared with the vendor’s recommendation. Formalin fixation–induced DNA damages were repaired using the NEBNext FFPE DNA Repair Kit (New England Biolabs). Sequencing libraries were prepared using the NEBNext Ultra II DNA Library Prep Kit for Illumina with NEBNext Unique Dual Index UMI Adaptors (New England Biolabs). After quantification and quality control with the Qubit HS dsDNA Kit (Qiagen) and TapeStation High Sensitivity D5000 Kit (Agilent), libraries were pooled equimolarly and loaded on a NextSeq2000 (Illumina) P2 or P3 flow-cell for 50 cycle, single-read sequencing with a target yield of 10 M bases per library. Sequencing metrics and genome-wide copy number burden results are summarized in Supplementary Table S1.

### Analysis of Low-Coverage Whole-Genome Sequencing Data

Raw intensities were converted to raw reads using bcl2fastq (v.2.20.0.422). Raw reads were trimmed using skewer (v.0.2.2) with the mean quality set to 10 and minimum length set to 25 bp in tail mode. Reads were aligned to GRCh38 (downloaded from: https://ftp.1000genomes.ebi.ac.uk/vol1/ftp/technical/reference/GRCh38_reference_genome/) using BWA-MEM (v.0.7.17.)-defining read groups and converting aligned reads to the BAM format using SAMtools (v.1.10). BAM files were then sorted by genomic coordinates using GATK SortSam (v.4.1.7), and BAM files from libraries run on a P3 dual-lane flow-cell were merged using SAMtools merge. Duplicate reads were marked using GATK MarkDuplicates with the optical duplicate pixel distance set to 2500. BAM files processed by GATK ValidateSamFile were analyzed for relative CNAs with QDNASeq (v.1.26.0) following standard recommendations in the vignette for normalization and segmentation of coverage data in R (v.4.0.2) using a custom 500 kilobase bin size mappability file available at https://github.com/bataibence/FL-sWGS-heterogeneity/blob/main/QDNAseq_bins_500kb_GRCh38_50bp_mappability_semmelweis.rds. Absolute copy numbers were determined using a custom R script available as a courtesy of George Cresswell. The script performs optimal purity and ploidy search based on creating a fit matrix for every relative alteration in the sample and every purity and ploidy option in order to find a local minima solution. Then, the optimal purity and ploidy solution is used for generating purity and ploidy-corrected absolute copy numbers from relative changes. The analysis was performed with ploidy restricted to 2 since hyperdiploid states are rare in follicular lymphoma. Purity search was performed between 0.2 and 1.0 and for samples without a local minima solution, the purity was set to 0.2 in order to detect CNAs. For patients with sequential samples, another analysis run was performed setting purity to 0.1 in order to recover potential subclonal alterations and alterations in low-purity samples, complemented with an aligned visual inspection of copy number bin profiles.

### Analysis of Identified Copy Number Alterations and Clinicopathological Data

Analysis of absolute copy numbers and statistical investigation were performed in RStudio Server (v.1.3.959) in R. Absolute copy numbers at or above 3 were considered amplifications, whereas absolute copy numbers at or below 1 were considered deletions. For the determination of cytoband level and chromosome arm-level alterations, we considered only those regions altered, where more than 50% of the respective regions’ bases were spanned by bins harboring a deletion or amplification. Distinctive alterations between patient groups were identified using the Fisher exact test. The prognostic value of CNAs was evaluated using Kaplan-Meier analysis and the log-rank test. Event-free survival (EFS) was defined as the time from diagnosis until clinical signs of recurrent disease, administration of antilymphoma therapy, or death of any cause, whichever occurred first. For all *P* values, we considered changes to be significant below .05. Visualization of data was performed using the ggplot (v.3.4.2) and ComplexHeatmap (v.2.6.2) packages.

## Results

### Clinicopathological Characteristics of the Patient Cohort

Clinical characteristics of the patient cohort are summarized in Table 1. The male-to-female ratio was 0.3, and median age at diagnosis was 60 years (range: 35-76 years). Most patients were diagnosed in stage T1a (16/20, 80%), whereas T1b (2/20, 10%) and T2a stages occurred less frequently (2/20, 10%). Clinical presentation was single or multiplex nodules or plaques occurring most commonly on the trunk (9/20, 45%) or the head and neck (8/20, 40%), whereas 2 patients were presenting with lesions on the upper (2/20, 10%) and 1 on the lower (1/20, 5%) extremities. In addition to surgical excision, radiotherapy was also administered in the first treatment line in 8 cases. Median follow-up time and EFS were 61 months (range: 13-157 months) and 23 months (range: 3-83 months), respectively. Disease recurrence was identified during the disease course in 15/20 patients (75%), affecting the same anatomical region in most of the cases (9/20, 45%), whereas distant skin recurrences occurred less frequently (5/20, 25%) and systemic spread was observed for only 1 case (1/20, 5%). The number of recurrences ranged from 1 to 3 (median 2) during the follow-up period.

**Table 1:** Clinical characteristics of the patient cohort

Patient ID	Sex	Age (y) at diagnosis	Stage at diagnosis^a^	Region at diagnosis^a^	First line treatment	EFS (mo)	Follow-up (mo)	Recurrence site	Number of recurrences	Samples analyzed
Case #1	Female	53	T2a	Head and neck	Surgical excision + radiotherapy	8	13	Local	1	Primary
Case #2	Female	52	T1a	Chest	Surgical excision + radiotherapy	45	152	Distant	3	Primary
Case #3	Female	66	T1b	Upper back	Surgical excision + radiotherapy	8	81	Local	1	Primary
Case #4	Female	57	T1a	Chest	Surgical excision	25	107	Local	1	Recurrence
Case #5	Female	60	T1a	Left upper arm	Surgical excision + radiotherapy	25	104	Distant	3	Primary and recurrence
Case #6	Female	63	T1a	Upper back	Surgical excision + radiotherapy	83	83	No		Primary
Case #7	Female	53	T1a	Upper back	Surgical excision	3	137	Local	2	Recurrence
Case #8	Female	67	T1a	Head and neck	Surgical excision	79	79	No		Primary
Case #9	Female	43	T1a	Upper back	Surgical excision	63	63	No		Primary
Case #10	Female	69	T1a	Head and neck	Surgical excision	34	72	Local	1	Primary
Case #11	Female	68	T1a	Chest	Surgical excision	14	157	Distant	3	2 recurrences
Case #12	Female	73	T2a	Upper back	Surgical excision	32	46	Systemic	1	Primary and recurrence
Case #13	Male	76	T1a	Head and neck	Surgical excision + radiotherapy	45	45	No		Primary
Case #14	Male	71	T1b	Head and neck	Surgical excision	16	34	Local	1	Recurrence
Case #15	Male	64	T1a	Head and neck	Surgical excision	5	52	Local	3	2 recurrences
Case #16	Female	57	T1a	Left upper leg	Surgical excision + radiotherapy	19	43	Distant	3	Recurrence
Case #17	Male	35	T1a	Head and neck	Surgical excision + radiotherapy	20	20	No		Primary
Case #18	Male	60	T1a	Upper back	Surgical excision + radiotherapy	36	57	Local	2	2 recurrences
Case #19	Female	52	T1a	Head and neck	Surgical excision	5	27	Local	1	Primary
Case #20	Male	45	T2a	Left lower arm	Surgical excision	15	59	Distant	2	Primary and 3 recurrences

a Stage and region was determined according to the TNM classification system for primary cutaneous lymphomas other than mycosis fungoides and Sézary syndrome (8).

Pathological characteristics of the 28 analyzed samples from the 20 patients are summarized in Table 2. Altogether, we analyzed 13 samples at primary diagnosis and 15 at recurrence, including sequential samples from 6 patients. Considering all samples, 13/28 (46%) presented with nodular, 12/28 (43%) mixed, and 3/28 (11%) samples with diffuse growth patterns. *IGH::BCL2* translocation status was evaluated by FISH in 15/28 samples and returned a positive result in only 1 sample (7%). Six of the 21 samples evaluated by FISH for 1p36 deletion were positive (29%).

**Table 2:** Pathological characteristics of patient samples

Patient ID	Sample analyzed	Region of biopsy	Recurrence site	Systemic treatment before sampling	Elapsed time from diagnosis (mo)	Growth pattern	CD10	BCL2	Ki67	IGH:BCL2 translocation	1p36 deletion FISH	IGH rearrangement
Case #1	Primary	Head and neck	-	No	0	Nodular	+	+	10%	-	-	Nonclonal
Case #2	Primary	Chest	-	No	0	Nodular	−	−	50%	-	-	Clonal
Case #3	Primary	Upper back	-	No	0	Mixed	−	−	40%	Failed	-	Nonclonal
Case #4	Recurrence	Chest	Local	No	27	Nodular	+	−	80%	-	+	No product
Case #5	Primary	Left upper arm	-	No	0	Mixed	+	−	30%	-	+	Nonclonal
Case #5	2nd recurrence	Head and neck	Distant	No	41	Nodular	−	−	15%	− (O)	+ (O)	Clonal
Case #6	Primary	Upper back	-	No	0	Nodular	+	−	10%	-	-	Clonal
Case #7	1st recurrence	Upper back	Local	No	30	Nodular	−	−	30%	− (O)	− (O)	Nonclonal
Case #8	Primary	Head and neck	-	No	0	Mixed	+	+	15%	-	-	Clonal
Case #9	Primary	Upper back	-	No	0	Mixed	+	−	25%	Failed	-	Clonal
Case #10	Primary	Head and neck	-	No	0	Nodular	+	−	5%	− (O)	+ (O)	Nonclonal
Case #11	2nd recurrence	Head and neck	Distant	No	100	Mixed	+	+	40%	-	+	Clonal
Case #11	3rd recurrence	Head and neck	Distant	No	106	Mixed	+	+	40%	− (O)	+ (O)	Clonal
Case #12	Primary	Upper back	-	No	2	Nodular	−	−	40%	-	-	Nonclonal
Case #12	1st recurrence	Axillary lymph node	Systemic	No	39	Nodular	+	+	NA	− (O)	− (O)	Nonclonal
Case #13	Primary	Head and neck	-	No	0	Mixed	+	−	NA	NA	+	Nonclonal
Case #14	1st recurrence	Head and neck	Local	No	16	Diffuse	−	−	80%	-	-	Clonal
Case #15	1st recurrence	Head and neck	Local	No	5	Nodular	+	−	20%	− (O)	− (O)	Clonal
Case #15	2nd recurrence	Head and neck	Local	No	12	Nodular	+	+	60%	-	-	Clonal
Case #16	1st recurrence	Left upper leg	Local	No	8	Mixed	+	+	60%	-	-	Clonal
Case #17	Primary	Head	-	No	0	Mixed	+/−	−	30%	-	-	Clonal
Case #18	1st recurrence	Upper back	Local	No	37	Nodular	+	+	15%	-	-	Clonal
Case #18	2nd recurrence	Upper back	Local	No	55	Nodular	+	+	30%	− (O)	− (O)	Clonal
Case #19	Primary	Head and neck	-	No	0	Mixed	+	+	20%	+	-	Clonal
Case #20	Primary	Left lower arm	-	No	0	Mixed	−	+	50%	− (O)	-	Nonclonal
Case #20	1st recurrence medial	Left upper leg	Distant	No	16	Diffuse	+	+	90%	− (O)	+	No product
Case #20	1st recurrence lateral	Left upper leg	Distant	No	16	Diffuse	+	+	80%	-	+ (O)	No product
Case #20	2nd recurrence	Left upper leg	Distant	Yes	50	Mixed	−	+	20%	− (O)	+	Nonclonal

(O), an other sample of the patient was evaluated.

Using *IGH* sequencing as part of the routine diagnostic workup, 15/28 (54%) samples showed clonal *IGH* rearrangement, 10/28 (36%) showed a nonclonal *IGH* repertoire, whereas 3/28 (11%) gave no evaluable product.

### Copy Number Alterations in Primary Cutaneous Follicle Center Lymphoma

**Figure 1:**
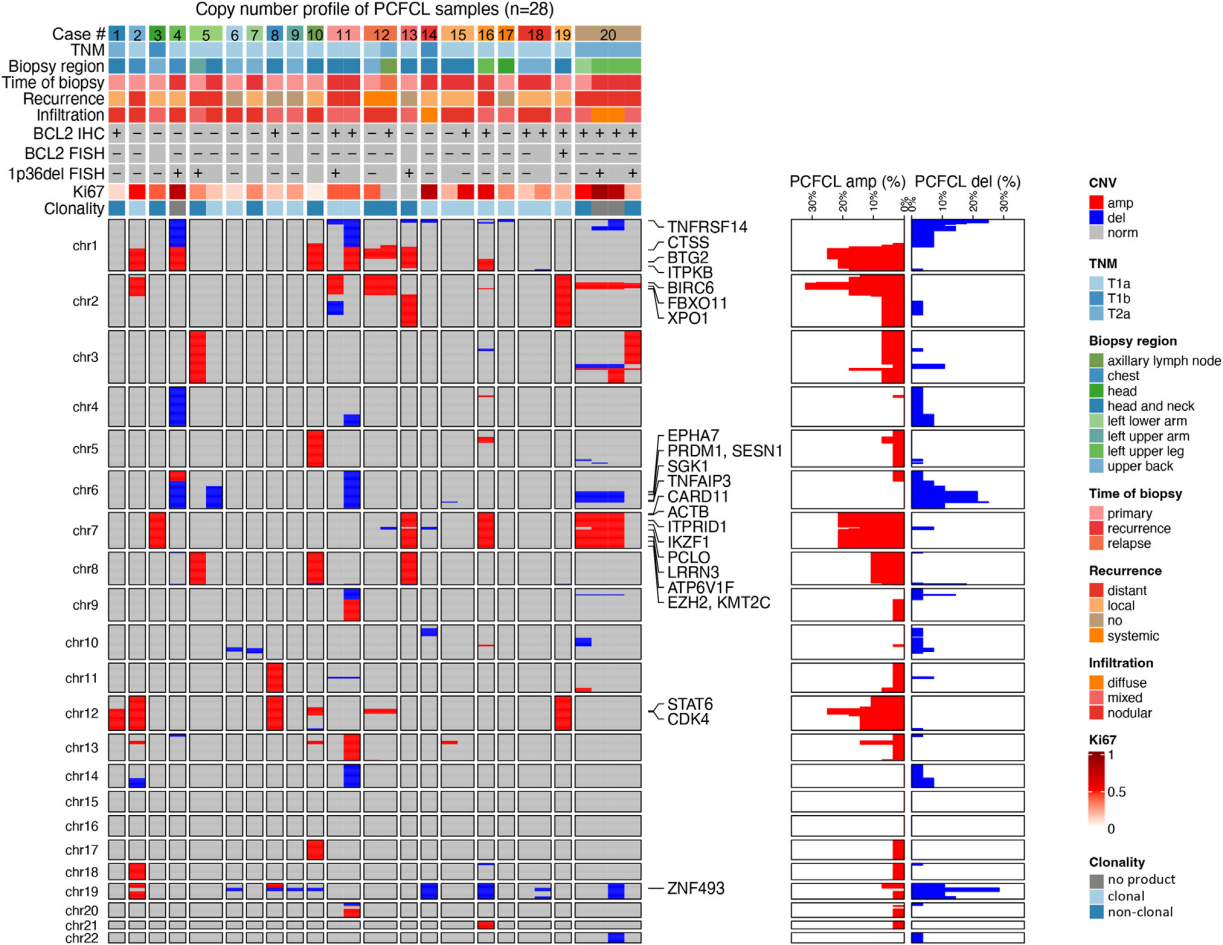
Copy number profile of all primary cutaneous follicle center lymphoma samples analyzed in the study (*n* = 28) displaying associated clinicopathological characteristics and frequency of copy number alterations. Genes in regions harboring a copy number alteration in at least 20% of the samples are displayed on the right side of the heatmap. amp, amplification; del, deletion; FISH, fluorescence in situ hybridization; IHC, immunohistochemistry; norm, normal; TNM, tumor, node, metastasis stage of cutaneous lymphomas other than mycosis fungoides and Sezary syndrome.

We performed lcWGS on 28 samples of 20 PCFCL patients. At least 1 CNA was detected in 26/28 (92.9%) samples with median of 4 CNAs (IQR: 2-8) per sample (Supplementary Table S1). The most frequently identified copy number losses at the cytoband level were the deletions of the 19p12-q13.11 region (28.6%), 1p36.33-p36.23 and 6q24.1 regions (25.0%), as well as the deletions in the 6q16.1-q23.3 regions (21.4%), whereas the most frequently identified copy number gains were spanning the 2p22.2-p15 (32.1%), 2p22.3 (28.6%), 12q13.13-q14.3 (25.0%), and 1q21.2-q25.3 (25.0%) regions (Fig. 1, Supplementary Fig. S1A and Table S2). Arm-level deletions were most frequently affecting the 6q, 19p, and 19q chromosome arms (all, 10.7%), whereas arm-level amplifications were present in the 1q, 7p, and 7q chromosome arms in 21.4% of all samples (Supplementary Fig. S1B). Amplifications were more often conferred by arm-level changes, whereas deletions were often focal. Focal amplification was detected in 59 of 508 (11.6%) altered cytobands, whereas focal deletion was observed in 126 of 273 (46.2%) altered cytobands (Supplementary Fig. S2, Tables S3 and S4).

Deletions of 1p36 were investigated by both FISH and lcWGS in 20 samples (Fig. 1). Of these, 13 samples provided a concordant negative result, whereas 4 samples had a concordant positive result. In 2 samples, only FISH was positive, whereas in 1 sample, a 1p36 deletion was solely identified using lcWGS. Overall, this resulted in a positive and negative predictive value of lcWGS for the 1p36 deletion of 80% and 87%, respectively.

Clonality assessment was successfully performed in 25/28 samples using *IGH* sequencing. Notably, in all 3 samples with no product during clonality analysis, multiple CNAs could be detected providing clear evidence of clonal proliferation. Furthermore, in all samples with a nonclonal *IGH* repertoire (*n* = 10), 1 (3/10, 30%) or multiple (7/10, 70%) CNAs were identified (Fig. 1).

### Comparison of the Copy Number Profiles of Primary Cutaneous Follicle Center Lymphoma and Nodal Follicular Lymphoma

**Figure 2:**
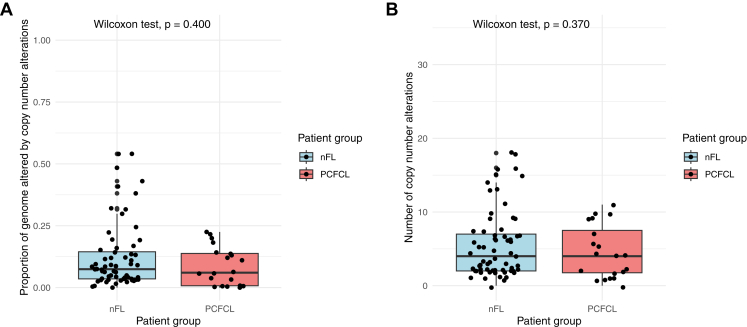
Comparison of copy number burden metrics and frequency of distinctive copy number alterations between nodal follicular lymphoma (nFL, *n* = 64) and primary cutaneous follicle center lymphoma (PCFCL, *n* = 20) samples including the first sample of every patient. (A) Comparison of proportion of genome altered by copy number changes between nFL and PCFCL. (B) Comparison of the number of identified copy number changes between nFL and PCFCL.

Subsequently, we compared the copy number profiles of PCFCL samples with data generated from a process-matched cohort of diagnostic, pretreatment nFL samples (*n* = 64), constituting of patients with limited stage disease (Ann Arbor I-II) in 13.5% and advanced stage disease (Ann Arbor III-IV) in 86.5% of cases. To remove the distortion effect of sequential samples in the PCFCL cohort, we performed the analysis including only on the first sample of each patient. Investigating copy number burden metrics, the median proportion of genome altered (PCFCL: 0.06, IQR: 0.01-0.14 vs nFL: 0.07, IQR: 0.04-0.14, *P* = .400, Wilcoxon test), as well as the median number of identified copy number changes, were similar between the 2 patient groups (PCFCL: 4, IQR: 2-8 vs nFL: 4, IQR: 2-7, *P* = .370, Wilcoxon test) (Fig. 2; Supplementary Fig. S3).

Our results indicated relatively similar copy number profiles of PCFCL and nFL (Fig. 4). Most frequent CNAs in both cohorts were deletions on 1p and 19p chromosome arms affecting *TNFRSF14* and *ZNF493*, respectively, and amplifications on 1q, 2p, and 12p chromosome arms spanning the coding regions of *CTSS*, *BIRC6*, *FBXO11*, *XPO1*, *STAT6*, and *CDK4*.

**Figure 3:**
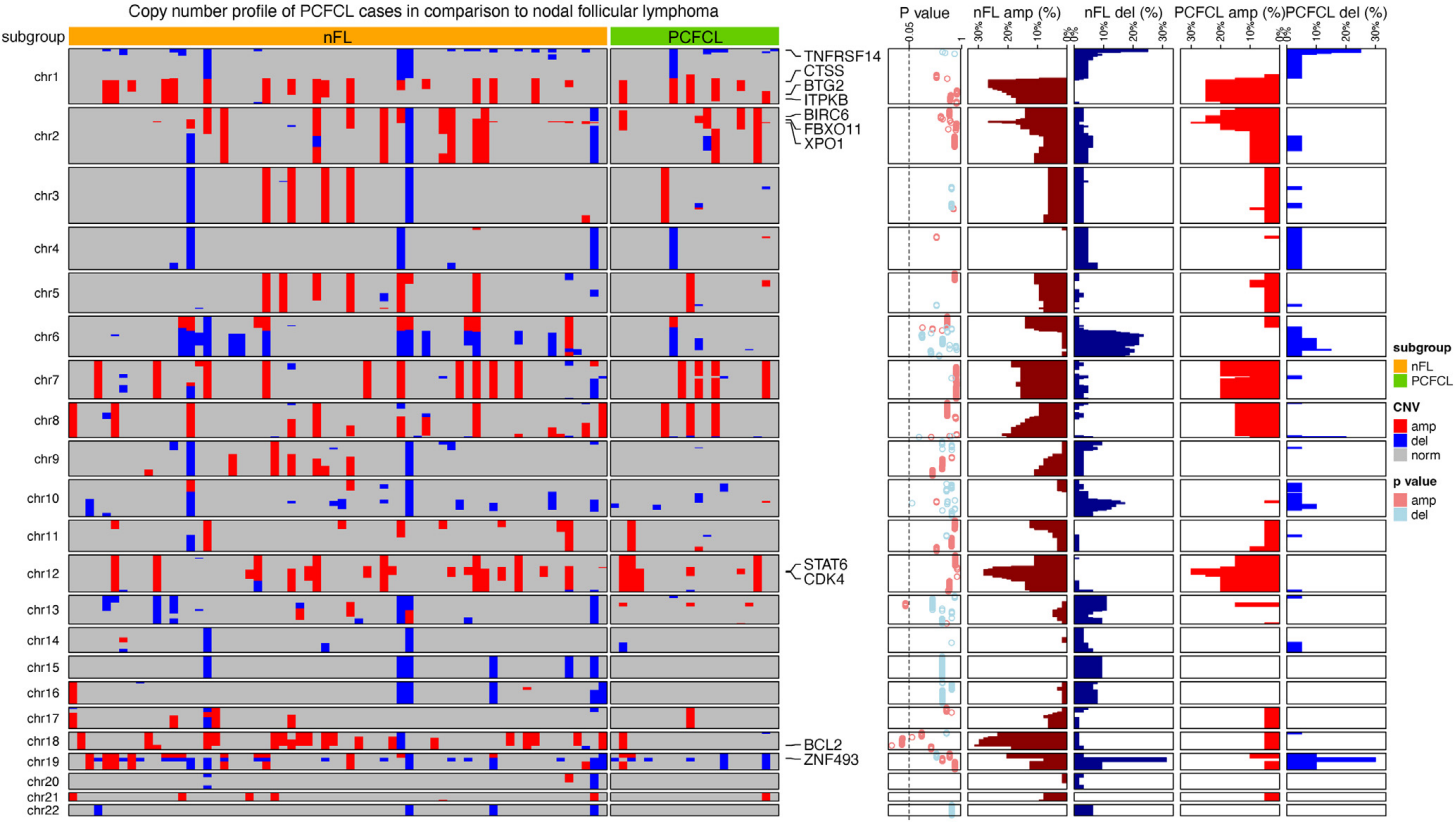
Copy number profile of primary cutaneous follicle center lymphoma (PCFCL) cases in comparison to a cohort of process-matched nodal follicular lymphoma (nFL) samples. The heatmap displays the copy number profile of nFL (*n* = 64) and PCFCL (*n* = 20) samples, highlighting the most frequently affected (frequency above 25% in any of the cohorts) genes on the right side of the heatmap. *P* values of the Fisher exact test for the corresponding cytoband are displayed on the dotplot on the right side of the heatmap for values below 1.0. Next to the *P* values, the frequencies of deletions (del) and amplifications (amp) are visualized separately for each group as bar plots.

Interestingly, of the most frequent alterations known in nFL, amplifications on chromosome 18q peaking at the 18q21.33 cytoband and affecting the locus of the *BCL2* oncogene showed significant enrichment in nFL samples (31.3% [20/64]), whereas it was detected only in 1 PCFCL sample (5.0% [1/20], *P* = .018, Fisher exact test) (Fig. 3; Supplementary Table S3). Significant enrichment of amplifications in the 13q14.11-q14.2 region was observed in PCFCL (PCFCL: 15.0% [3/20] vs nFL: 1.6% [1/64], *P* = .040, Fisher exact test) spanning the coding region of *FOXO1*. After including all PCFCL samples in the analysis to account for potential temporal heterogeneity, all identified distinctive regions remained significantly enriched in either subgroup. Additionally, the absence of deletions in the 10q23.32 cytoband was observed as a distinctive feature between PCFCL and nFL samples (PCFCL: 0.0% [0/28] vs nFL: 17.2% [11/64], *P* = .017, Fisher exact test; Supplementary Table S4).

Despite the low prevalence of *IGH::BCL2* translocation and 18q21.33 amplification commonly leading to BCL2 overexpression in nFL, BCL2 expression was still observed in 50% (14/28) of PCFCL samples by immunohistochemistry using a conservative BCL2 cutoff above 80%, with further 3 cases harboring a BCL2 expression between 50% and 80% considered negatives. Scrutinizing the differences between the CNA profiles of PCFCL samples showing BCL2-positive vs -negative phenotypes, we did not observe significantly enriched alterations in BCL2-positive cases. Interestingly, 2p22.2-p15 amplifications encompassing the *XPO1* and *REL* genes, potentially enhancing the BCL2 expression via the NF-kB pathway, were detected in more BCL2-positive samples, although this difference was not statistically significant (14.3% [2/14] vs 50.0% [7/14], *P* = .103; Supplementary Table S4).

### Prognostic Significance of Copy Number Alterations in Primary Cutaneous Follicle Center Lymphoma

In order to identify CNAs potentially associated with disease recurrence, we investigated the differences between the copy number profiles of unrelated primary and recurrence samples. Median proportion of genome altered (primary: 0.11 [IQR: 0.03-0.14] vs recurrence: 0.04 [IQR: 0.01-0.10], *P* = .490) and the number of CNAs (primary: 4 [IQR: 2-7] vs recurrence: 4 [IQR: 2-8], *P* = 1.000) showed no difference between the patient groups (Supplementary Fig. S4). Investigating potentially distinctive regions, significant enrichment of 1p36.23-p36.22 deletions was found in the recurrence samples spanning the coding region of *MTOR* (7.7% [1/13] vs 57.1% [4/7], *P* = .031; Supplementary Table S3).

Stratifying patients by distant cutaneous recurrence, omitting the case developing systemic propagation from the analysis proportion of genome altered (localized: 0.04 [IQR: 0.01-0.13] vs distant: 0.13 [IQR: 0.12-0.14], *P* = .190) and number of CNAs (localized: 2 [IQR: 1-6] vs distant: 10 [IQR: 4-10], *P* = .044) was moderately higher in patients developing distant cutaneous spread (Supplementary Fig. S5). Amplifications on the 2p arm were significantly enriched peaking in the 2p15 cytoband coding *XPO1* (7.1% [1/14] vs 80.0% [4/5], *P* = .006) and the neighbor 2p22.3-p16.1 cytobands coding *REL* (7.1% [1/14] vs 60.0% [3/5], *P* = .037; Supplementary Table S3; Fig. S5).

**Figure 4:**
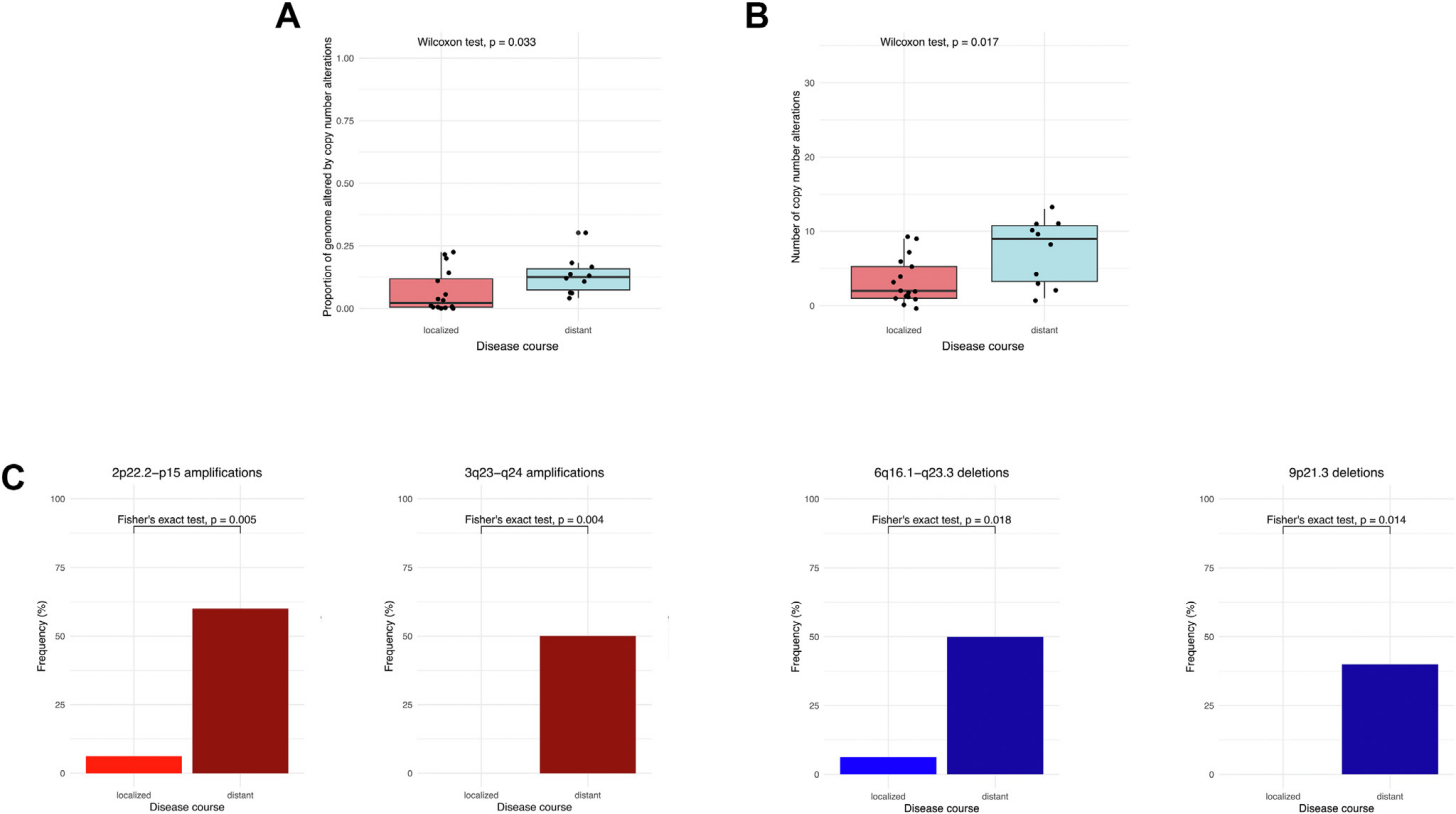
Distribution of copy number burden metrics and alterations in distinctive regions between primary cutaneous follicle center lymphoma (PCFCL) patients showing localized disease course compared with patients developing distant cutaneous spread or systemic disease. (A-B) Proportion of genome altered and the number of identified copy number alterations were significantly higher in patients with distant disease spread suggesting increased genomic instability. (C) Including all samples, 2p22.2-p15 and 3q23-q24 amplifications, as well as 6q16.1-q23.3 and 9p21.3 deletions were detected as potentially prognostic biomarkers of the disease course in PCFCL.

Analyzing all PCFCL samples, copy number burden was significantly higher in samples of patients’ developing distant cutaneous spread both in terms of proportion of genome altered (localized: 0.02 [IQR: 0.01-0.12] vs distant: 0.13 [IQR: 0.07-0.16], *P* = .033) and number of CNAs (localized: 2 [IQR: 1-5] vs distant: 9 [3-11], *P* = .017) pointing to higher genomic instability in samples more readily involving distant disease sites (Fig. 4). Additionally, the enrichment of 12q amplifications was also observed in primary samples peaking in the 12q13.13-q14.3 region coding *STAT6* and *CDK4* (46.2% [6/13] vs 6.7% [1/15], *P* = .029), whereas 3q23-q24 amplifications (0.0% [0/16] vs 41.7% [5/12], *P* = .008) and 9p21.3 deletions (0.0% [0/16] vs 40.0% [4/10], *P* = .014) covering *CDKN2A* and *CDKN2B* in addition to deletions of 6q16.1-q23.3 (6.3% [1/16] vs 50.0% [5/10], *P* = .018) covering *PRDM1* and *TNFAIP3* were more frequently observed in patients developing distant cutaneous spread (Fig. 4; Supplementary Table S4). We analyzed the prognostic significance of recurrent CNAs using the Kaplan-Meier method; however, EFS was not associated with alterations in any of the investigated cytobands based on the log-rank test (Supplementary Table S3).

### Evolution of the Copy Number Profile of Primary Cutaneous Follicle Center Lymphoma During the Clinical Course

Comparing the copy number profiles of sequential tissue samples from 6 patients, overlapping alterations were observed in 3 patients during the disease course with additional emerging and diminishing alterations underlining the role of spatiotemporal heterogeneity in the pathogenesis of PCFCL (Fig. 1). In 2 patients, there were no CNAs in one of the samples potentially pointing to a flat copy number profile. In case #5, the primary and recurrence samples harbored different alterations, including chromosome 3 and 8 amplifications and 1p36 deletion in the primary sample, but an isolated 6q deletion in the recurrence sample (Fig. 1). Additionally, the primary and recurrence samples showed marked differences in phenotype and *IGH* repertoire usage. The primary sample showed mixed nodular and diffuse proliferation with BCL6, CD10, and BCL2 positivity and a polyclonal *IGH* profile, whereas the recurrence sample showed nodular, BCL6-positive, CD10- and BCL2-negative, *IGH* monoclonal proliferation, suggesting the parallel development of 2 separate malignant clones, with the second one expanding at recurrence.

With regard to the clinical outcome, 2 patients with available sequential samples displayed an unfavorable clinical course, 1 with the development of systemic spread (case #12) or distant skin recurrence and underlying soft tissue infiltration requiring chemoimmunotherapy (case #20).

**Figure 5:**
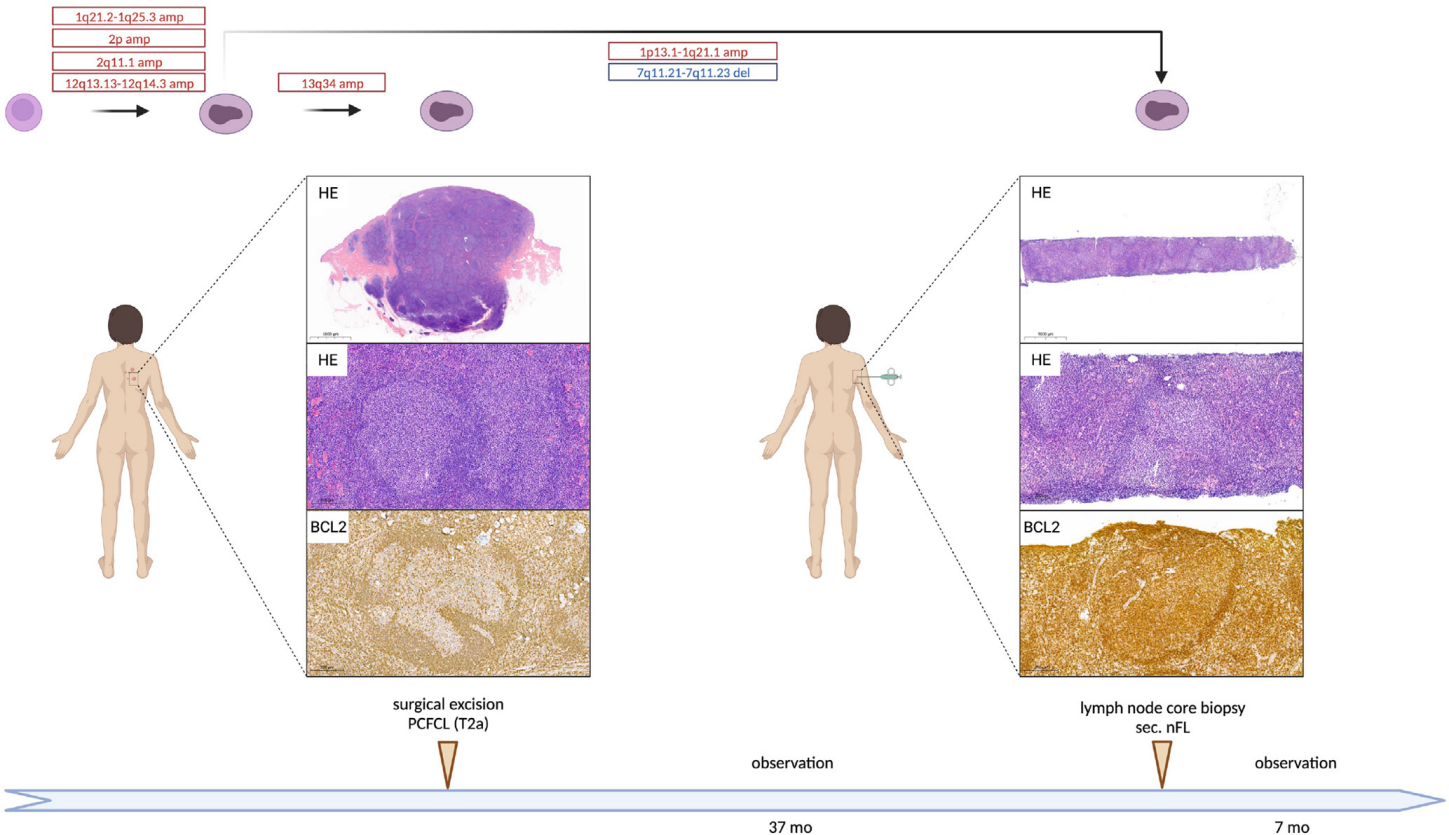
Comprehensive overview of disease course in case #12 developing systemic propagation to secondary nodal follicular lymphoma (nFL). Case #12 was diagnosed with stage T2a PCFCL at the age of 73, which harbored a 1q21.2-q25.3, 2p arm, 2q11.1, 12q13.13-q14.3, and 13q34 amplification as later revealed by low-coverage whole-genome sequencing. After 32 months of observation, right axillary lymphadenopathy was detected. Low-coverage whole-genome sequencing uncovered the previously identified amplifications in the background of systemic propagation except for the 13q34 amplification with newly acquired alterations such as a 1p13-q21.1 amplification and a 7q11.21-q11.23 deletion.

Case #12 had a history of erythematous plaques on her back for 3.5 years, when surgical excision of a plaque and a cutaneous nodule was performed. Histology revealed PCFCL with nodular growth pattern, with CD10, BCL2-negative centrocytic infiltrate (Fig. 5; Supplementary Fig. S6). Staging found no evidence of systemic involvement. Thirty-seven months later, multiplex enlarged lymph nodes were detected in the right axillary region. Pathological examination of the core biopsy revealed infiltration of CD10- and BCL2-positive nodular centrocytic infiltration consistent with classical follicular lymphoma (Fig. 5; Supplementary Fig. S6). Spontaneous regression of the lymph nodes was observed on ultrasonography during the watch and wait period, and the patient does not have any lymphoma related symptoms at the end of follow-up. In the primary sample, lcWGS recovered amplifications in the 1q21.2-q25.3, 2p arm, 2q11.1, 12q13.13-q14.3, and 13q34 regions. The previously identified amplifications were uncovered in the lymph node sample except for the 13q34 amplification with newly acquired alterations including a 1p13-q21.1 amplification and a 7q11.21-q11.23 deletion (Fig. 5).

**Figure 6:**
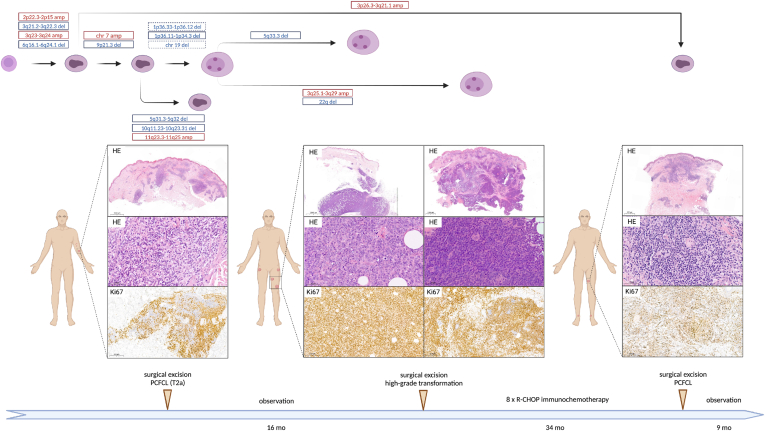
Comprehensive overview of disease course in case #20 developing high-grade recurrence requiring chemoimmunotherapy. Low-coverage whole-genome sequencing revealed 2p22.3-p15 and 3q23-q24 amplifications, as well as 3q21-q22.3 and 6q16-q24.1 deletions in all 4 samples of the patient possibly representing the earliest events in lymphomagenesis, with the clone later acquiring lymphoma specific alterations such as 1p36 deletion potentially facilitating clinical and morphological progression. Systemic chemoimmunotherapy successfully eliminated the expanded highly proliferating subclone, but the disease resembling the earliest common precursor recurred as a low-grade process.

Case #20 presented with a subcutaneous nodule on his lower left arm, accompanied by erythematous papules on his left upper arm, showing mixed nodular and diffuse infiltration of centrocytes consistent with PCFCL (Fig. 6; Supplementary Fig. S6). Sixteen months later, the disease recurred as a fast-growing subcutaneous nodule in the medial part of the left thigh surrounded by a group of small papules, as well as erythematous plaques on the lateral side of the hip. Histological examination of the medial nodule revealed subcutaneous diffuse infiltration of mostly centroblasts accompanied by a high number of mitotic and apoptotic figures, whereas the lateral lesion showed monotonous, diffuse, large centrocytic infiltrate with blastoid morphology in the dermis. Leg-type DLBCL was excluded in both cases based on MUM1 negativity and lack of immunoblasts, the presence of centrocytes, and negative results for *BCL2*, *BCL6*, and *MYC* translocations by FISH (Supplementary Table S5). Positron emission tomography–computed tomography restaging described increased subcutaneous and deep soft tissue 18-fluorodeoxyglucose uptake near the lesions with no evidence of further systemic involvement. Suspected soft tissue involvement indicated chemoimmunotherapy treatment resulting in complete morphometabolic remission. Thirty-four months later, a relapse occurred in the left thigh and around both ankles. Surgical excision from the left thigh revealed nodular centrocytic infiltrate with BCL6, BCL2 co-expression, and low proliferation rate, consistent with classic PCFCL resembling the primary sample. Since the surgical excision, the patient has been in complete remission.

In the respective primary sample, lcWGS revealed 2p22.3-p15 and 3q23-q24 amplifications, as well as 3q21-q22.3 and 6q16-q24.1 deletions that were detected throughout the clinical course, in addition to chromosome 7 amplification and 9p21.3 deletion common to the clone leading to high-grade relapse, morphologically resembling the systemic high-grade B-cell lymphoma diagnostic category. Amplification of 11q23.3-q25 and 5q31.3-q32, as well as deletion of 10q11.23-q23.31, was specific to the primary sample (Fig. 6). At first relapse, both morphologically high-grade tumor samples acquired a 1p36.33-p36.12 deletion and a chromosome 19 deletion, albeit these were subclonally detected in the medial lesion, and a 1p36.12-p34.3 deletion that was observed clonally in both samples. Furthermore, in the medial lesion, a 5q33.3 deleted subclone, whereas in the lateral lesion, a 3q25.1-q29 amplified and 22q deleted subclone expanded, pointing to spatially separated evolution. Systemic chemoimmunotherapy successfully eliminated the expanded high-grade subclone, but the disease recurred resembling the earliest common precursor with predominantly small centrocytic infiltration harboring an additional 3p26.3-q21.1 amplification (Fig. 6).

## Discussion

PCFCL is a rare and indolent disease with a partly unexplored genetic background. Recently, high-throughput sequencing studies identified a lower prevalence of somatic variants in epigenetic modifiers in the disease including *KMT2D*, *CREBBP*, and *EZH2* than that observed in nFL, but a similar prevalence of mutations in immunoregulatory genes, including *TNFRSF14*, *STAT6*, *FOXO1*, and *TNFAIP3*.^4^^,^^6^ Somatic variants in epigenetic modifiers and *IGH::BCL2* rearrangement have recently been integrated into a risk prediction algorithm for differentiating PCFCL from secondary cutaneous spread of nFL and PCFCL with later systemic propagation, highlighting similar dependence on epigenetic modification and BCL2 signaling.^6^ These alterations are probably less important in PCFCL showing a clinical course restricted to the skin.

Despite emerging data on short variants, genome-wide copy number profiles are only available for 54 PCFCL patients from 6 different studies using array CGH.^6^^,^^15^, ^16^, ^17^, ^18^, ^19^ These studies compared PCFCL with secondary cutaneous spread of nFL for differential diagnosis or PCFCL with large cells to primary cutaneous DLBCL (PCDLBCL), leg-type to uncover the prognostic significance of alterations and confirm the classification. Notably, a subset of these studies was either performed before the united WHO-EORTC classification of cutaneous lymphomas in 2005 or biased toward PCFCL large cells, sometimes making interpretability of data difficult.^1^

Using lcWGS, we identified CNAs in 26 of 28 PCFCL samples from 20 patients, with the most frequent deletions affecting chromosome arms 1p, 6q, and chromosome 19, and amplifications emerging on 1q, 2p, and 12q, which altogether is in line with previous data from cutaneous B-cell lymphomas^6^^,^^15^, ^16^, ^17^ and constitute a similar pattern to that of nFL.^20^^,^^21^ Notably, in our cohort, focal and gross 6q deletions were identified in up to 14.3% and 10.7% of the samples, respectively, which has been previously implicated as a distinctive feature of leg-type PCDLBCLs compared with PCFCL large cells in a patient cohort with similar size.^18^

We systematically compared the copy number distribution of PCFCL to a process-matched cohort of nFL cases, which revealed significant enrichment of amplifications on the 18q chromosome arm in nFL, peaking at 18q21.33 covering *BCL2*, whereas 13q14.11-q14.2 amplifications involving *FOXO1* were enriched in PCFCL. Amplifications on chromosome 18 were described to be present in around 25% of nFL cases and provide a complementary genetic basis for the activation of *BCL2* both with and without *IGH::BCL2* rearrangement.^20^^,^^22^ Additionally, amplifications on chromosome 18 can play a role in the activation of *MALT1* and *PMAIP1*.^23^ Comparing 9 PCFCL and 13 leg-type PCLBCL cases, Hallermann and colleagues have shown that chromosome 18 amplifications were the most common CNAs across all cutaneous B-cell lymphomas in their cohort (8/22); however, it was exclusively limited to PCDLBCL and were absent from PCFCL cases.^17^ Our results underline the fact that *BCL2* amplification has a minor role in PCFCL pathogenesis in contrast to nFL and provide evidence in line with literature data that amplification on chromosome 18 can be analyzed for differential diagnostic purposes, potentially both differentiating PCFCL from nFL and PCDLBCL. Based on our results, 2p22.2-p15 amplification had a higher frequency in PCFCL samples displaying detectable BCL2 expression. The amplification potentially leads to *REL* overexpression and thus activation of the NF-kB pathway,^24^ which may enhance the expression of BCL2 in the absence of *IGH::BCL2* translocation or 18q21.33 amplification.

Amplification of the 13q14.11-q14.2 region, described in 15.0% of our cohort, has not yet been described as a recurrent CNA in lymphomas. The most reasonable candidate gene for the amplified region would be *FOXO1*, functioning as a transcriptional regulator and known to harbor recurrent oncogenic hotspot mutations in lymphomas enabling nuclear retention and avoidance of cytoplasmic inactivation.^25^

Investigating the role of CNAs in disease progression revealed a higher prevalence of 1p36.23-p22 deletions in recurrence samples, and these were more frequent in cases developing distant disease spread during the disease course. Deletions of this region were not previously implicated in the pathogenesis of lymphomas. In all but 1 case, it occurred together with the p terminal deletion of 1p36; however, its emergence as a sole event in patient #16 points to a potential role in pathogenesis. The region encompasses the coding sequences of *MTOR*, *TNFRSF8* (*CD30*), and *TNFRSF1B*, which are described to have an activating role in a majority of other lymphomas,^26^, ^27^, ^28^ potentially suggesting a pleiotropic effect of these genes in lymphomagenesis. This has already been described regarding TNFRSF8, which has subtype-specific effects on the growth of T-cell and B-cell lymphoma cell lines,^29^ as well as mTOR activity, which is restricted to ABC DLBCL-s in most of the cases.^30^

Disease recurrence in PCFCL is mainly localized to the surgical bed or anatomic region.^31^ Distant cutaneous and extracutaneous spread can increase subjective disease burden and suggest different biological backgrounds of disease spread. Previously, a higher number of genomic imbalances was observed in PCDLBCL than in PCFCL large cells.^16^^,^^17^ However, its prognostic role has never been investigated in PCFCL. In our series, both proportion of the altered genome and number of CNAs were higher in patients developing distant spread pointing to increased genomic instability in tumor samples of these patients. Investigating the role of distinct CNAs in disease propagation revealed that distant recurrence was highly associated with 2p amplifications peaking at the 2p15 cytoband, present in 4/5 patients at their first presentation and in 6/10 samples of patients developing distant recurrence. Furthermore, 2p amplifications were present in all samples of cases #12 and #20, suggesting the acquisition of 2p amplification in the earliest common progenitor leading to systemic involvement and soft tissue infiltration with high-grade morphology. The significantly enriched regions from 2p22.3-p15 are spanning the coding regions of *XPO1*, *REL*, *FBXO1*, and *BIRC6*, respectively. An important target of the region is *REL* in 2p16.1, which is a transcriptional regulator as a subunit of NF-kB harboring a central role in BCR signaling and malignant transformation. In early studies investigating nFL, *REL* amplifications were described only in cases undergoing high-grade transformation,^32^^,^^33^ but this was refined in later studies, also reporting amplifications in low-grade cases, yet with a slightly lower prevalence.^21^^,^^34^ Amplifications involving the locus of *REL* on 2p have been described in 2 studies as the most prevalent CNA in PCFCL with large cells, similar to our case #20^17,18^ but were absent from other studies.^15^^,^^16^^,^^19^ Another important target in this region is *XPO1* on 2p15, in which the p.E571K activating mutation has been described in classical Hodgkin lymphoma and primary mediastinal B-cell lymphoma with a prevalence of 25%.^35^ Activation of XPO1 leads to abnormal nuclear export resulting in the abnormal intracellular distribution of DNA damage control proteins and cell cycle regulators. Although recurrent hotspot mutations were absent in other germinal center lymphomas, recurrent amplifications have been described in DLBCL and PMBL, where those correlated with increased levels of the respective mRNA.^35^^,^^36^ Most probably, the growth-stimulating potential of amplifications covering the 2p16.3-p15 region is a cumulative effect of *REL* and *XPO1* activation, which highlights the role of this CNA in disease propagation and can have an important role in determining the disease course of PCFCL. Including all samples, 3q23-q24 amplification and 6q16.1-q23.3 deletion, as well as 9p21.3 deletion, were more frequently observed in samples from PCFCL patients developing distant recurrence. Amplifications of 3q have only been described in one of the seminal studies and were restricted to leg-type DLBCL cases.^15^ Deletions of the 6q chromosome arm were specific to PCDLBCL in a previous study comparing PCDLBCL with PCFCL, large cells, potentially explaining the increased tendency of distant spread in samples harboring this alteration. 6q deletions lead to the loss of crucial tumor-suppressor genes and immune regulators in the pathogenesis of mature lymphoid malignancies including *PRDM1*, *SGK1*, and *TNFAIP3*.^18^ Deletions of 9p21.3 were thought to be characteristic of leg-type DLBCL and conferring dismal prognosis.^16^^,^^18^^,^^37^ The exclusive occurrence of 9p21.3 deletion in PCDLBCL leg-type was described in cohorts with limited case numbers encompassing 6/6 and 5/12 9p21.3 deletion-positive PCDLBCL, as well as 0/4 and 0/19 9p21.3 deleted PCFCL cases.^16^^,^^18^ Using a higher resolution multiplex ligation-dependent probe amplification method, reanalysis of a subset of previously 9p21.3 negative cases in one of the studies revealed a single PCFCL case harboring a hemizygous 9p21.3 deletion.^18^^,^^37^ We identified 9p21.3 deletions in 4 of 28 samples from 2 patients. Case #11 acquired 9p21.3 deletion at third recurrence affecting the head and neck regions and showing small cell morphology. Case #20 had a detectable 9p21.3 deletion in the primary sample from the left lower arm, which later recurred in the legs as a fast-growing thick subcutaneous tumor with large cell morphology, 80% to 90% cell proliferation rate, and soft tissue involvement. Intriguingly, the 9p21.3-deleted subclone was eliminated by chemoimmunotherapy, and it was undetectable from the last recurrence sample with small cell morphology.

For the first time, we investigated spatiotemporal dynamics of genome-wide CNAs in PCFCL patients using consecutive samples from 6 patients, with 3 patients showing an overlapping copy number profile. Interestingly, clonally unrelated relapses were also detected in case #5.

Progressive clinical course occurred in 2 of the 6 cases. During disease propagation, expansion of different subclones was observed in both cases, with unique CNAs found in all samples pointing to remarkable evolutionary plasticity of CNAs in PCFCL. In case #12 showing systemic progression, the common precursor harbored an arm-level 2p and focal 1q and 12q amplifications, which have been known to play an established role in nFL pathogenesis^21^ and additionally acquired a focal gain in the centromeric region of chromosome 1, as well as a focal 7q11.21-q11.23 deletion during propagation to nFL. In case #20, focal deletion of 6q16.1-q24.1 found in the common precursor was previously associated with shorter overall survival in nFL^21^ and with the diagnosis of PCDLBCL.^19^ During disease propagation, a subclone harboring a 9p21.3 deletion previously associated with high-grade transformation of nFL and also a characteristic feature of PCDLBCL expanded, which was followed by the acquisition of a 3q25.1-q29 amplification detected in the sample with the most aggressive morphology, potentially further supporting the role of these genetic changes in PCFCL progression.^21^ Notably, the copy number profile of the case developing systemic spread included CNAs significantly associated with distant cutaneous spread in our analysis. However, as this observation is based only on a single case with systemic spread, it remains to be further investigated whether there is a link in pathogenesis between cases showing distant cutaneous spread and systemic propagation. Additionally, the wide morphological spectrum of PCFCL is well represented by this relapsing case in parallel with clonal evolution of the disease. In addition, it clearly confirms the recent classification, as these highly proliferative diffuse large cell tumors with germinal center B-cell phenotype belong to the spectrum of PCFCL.

It is important to note that this study has some intrinsic limitations. The low number of patients due to rarity of the disease makes prognostic evaluation of the data underpowered for subtle differences in our cohort. A further drawback is the missing data for sex chromosomes, which can be ambiguous when performed based on solely depth of coverage data and thus an intrinsic weakness of lcWGS, which remains to be unexplored by other molecular methods.

Herein, we interrogated the genome-wide copy number profile of PCFCL performing lcWGS for the first time in PCFCL and nFL. Based on our results, the copy number profile of PCFCL is only slightly different from that of nFL, with the notable exception of the scarcity of 18q amplifications including 18q21.33, which covers the *BCL2* locus. This further highlights its potential utility in differential diagnosis of PCFCL and secondary cutaneous infiltration of nFL. Our results point to higher genomic instability in patients developing distant disease spread. We further deciphered the role of 2p16.3-p15 amplifications in the disease course of PCFCL, which could be an early prognostic marker in the future for the prediction of distant disease spread. Deletions of 9p21.3 and 6q were recurrently identified in a subset of the PCFCL patients, refining data from previous studies reporting the exclusive occurrence of these CNAs in PCDLBCL in smaller cohorts. For the first time, we analyzed the temporospatial plasticity of PCFCL investigating serial patient samples highlighting evidence of branching evolutionary processes and spatial differences in the distribution of CNAs leading to disease propagation.
