# Supplementary material for: Profiling of Copy Number Alterations Using Low-Coverage Whole-Genome Sequencing Informs Differential Diagnosis and Prognosis in Primary Cutaneous Follicle Center Lymphoma
Source: Mod Pathol. 2024 May;37(5):100465. doi: 10.1016/j.modpat.2024.100465 (PMC11092316; doi:10.1016/j.modpat.2024.100465)
Supplement: Supplementary Data [file mmc1.pdf]

Supplementary figure 1.

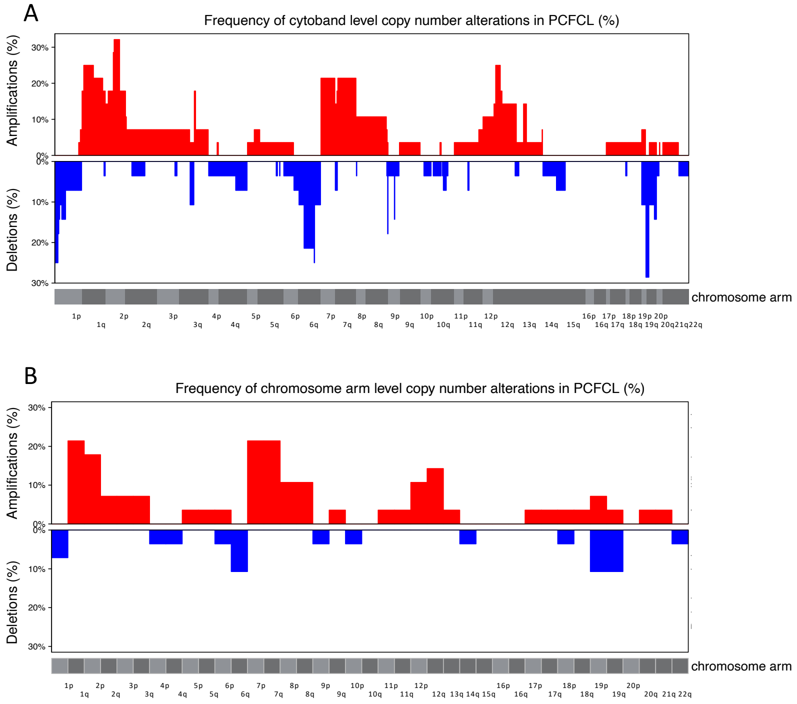

Supplementary figure 1. *Frequency of copy number alterations in primary cutaneous follicle center lymphoma. A) Frequency histogram of copy number alterations at cytoband resolution in PCFCL. Only those copy number changes were considered where at least 50% of the cytoband was covered by amplified or deleted segments. B) Frequency histogram of copy number alterations at the chromosome arm level. Only those copy number changes were considered where at least 50% of the chromosome arm was covered by amplified or deleted segments.*

Supplementary figure 2.

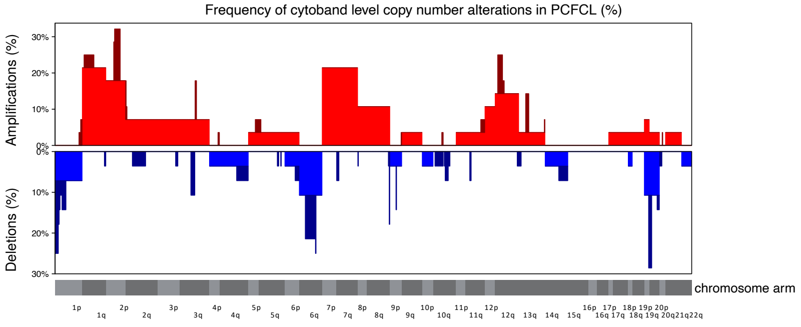

Supplementary figure 2. *Frequency of copy number alterations in primary cutaneous follicle center lymphoma differentiating focal (only observed as cytoband level change) and gross (observed at the chromosome arm level) alterations. Gross amplifications spanning cytobands are displayed with blue, while focal amplifications are displayed with darkblue. Gross and focal deletions are displayed with red and darkred, respectively.*

Supplementary figure 3.

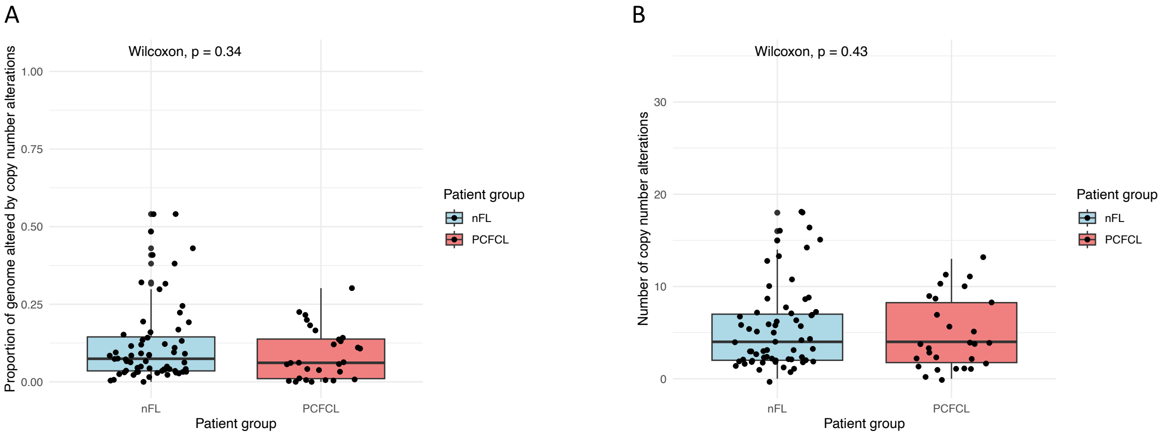

Supplementary figure 3. *Comparison of copy number burden metrics between nodal follicular lymphoma (nFL, n=64) and primary cutaneous follicle center lymphoma (PCFCL, n=28) samples. A) Comparison of proportion of genome altered by copy number changes between nFL and PCFCL. B) Comparison of the number of identified copy number changes between nFL and PCFCL.*

Supplementary figure 4.

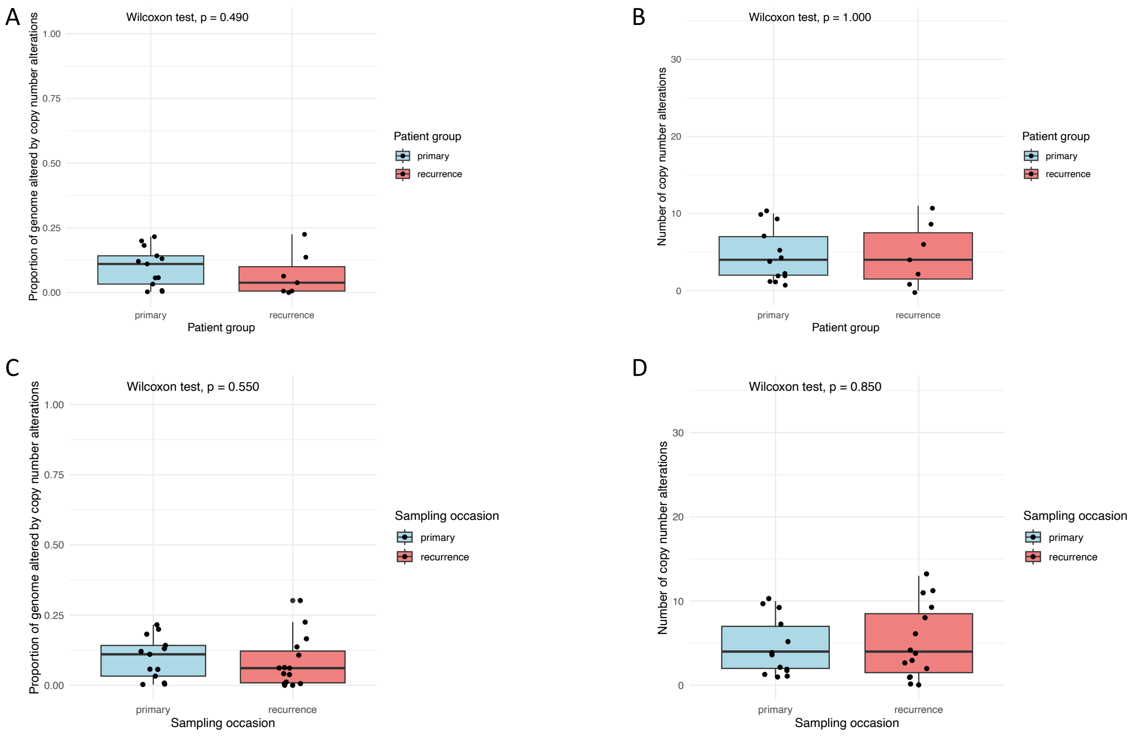

Supplementary figure 4. *Comparison of copy number burden metrics between primary and recurrence primary cutaneous follicle center lymphoma (PCFCL) samples. A-B) Proportion of genome altered and number of identified copy number alterations comparing the first sample of every patient. C-D) Proportion of genome altered and number of identified copy number alterations including all patient samples in the analysis.*

Supplementary figure 5.

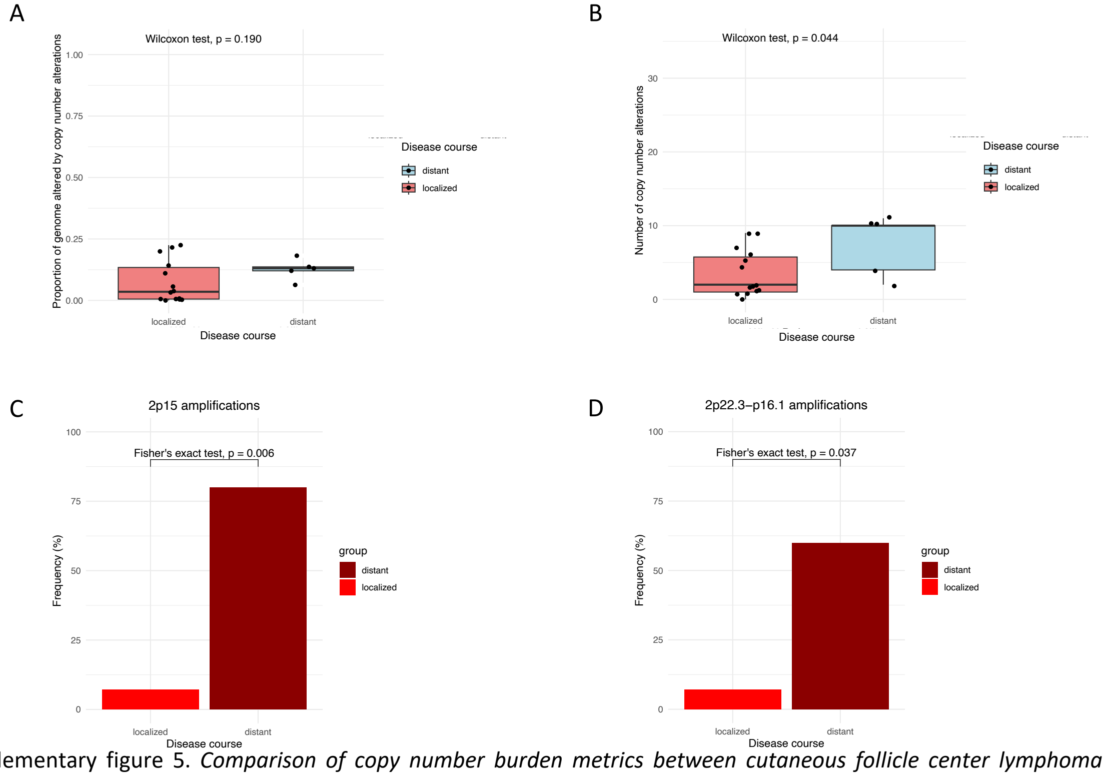

Supplementary figure 5. *Comparison of copy number burden metrics between cutaneous follicle center lymphoma (PCFCL) patients showing a localized disease course or distant cutaneous spread. A-B) Proportion of genome altered and number of identified copy number alterations comparing the first sample of every patient. C-D) Distinctive copy number alterations comparing the first sample of every patient.*

Supplementary figure 6.

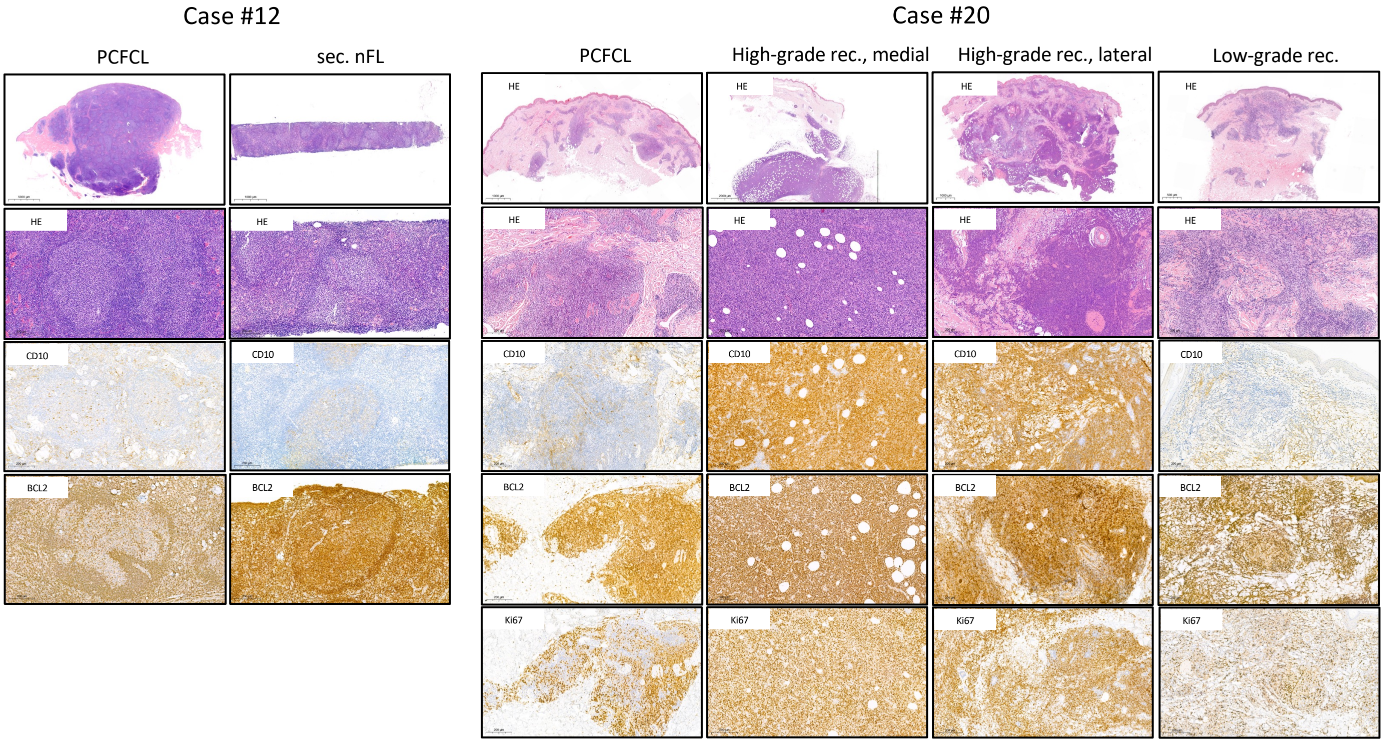

Supplementary figure 6. *Histopathological characteristics of patient samples in two representative cases with multiple tumor samples available for analysis during the disease course. Case #12 presented with CD10-, BCL2- nodular proliferation. Secondary nodal involvement retained nodular growth pattern, but the immunophenotype of tumor cells changed to CD10+ and BCL2+ as is frequently seen in nFL. Case 20# presented as PCFCL showing CD10-, BCL2+ mixed nodular and diffuse proliferation with predominantly small cell morphology. High-grade proliferation at first recurrence showed diffuse proliferation of CD10+, BCL2+ large centrocytes with a markedly elevated Ki67 expression at both sites. After immunochemotherapy treatment the disease recurred as a low-grade, nodular proliferation resembling the primary tumor sample.*
