# Supplementary material for: Profiling of Copy Number Alterations Using Low-Coverage Whole-Genome Sequencing Informs Differential Diagnosis and Prognosis in Primary Cutaneous Follicle Center Lymphoma
Source: Mod Pathol. 2024 May;37(5):100465. doi: 10.1016/j.modpat.2024.100465 (PMC11092316; doi:10.1016/j.modpat.2024.100465)
Supplement: Supplementary Data [file mmc2.docx]

**Supplementary material**

**Profiling of copy number alterations using low-coverage whole-genome sequencing informs differential diagnosis and prognosis in primary cutaneous follicle center lymphoma**

Bence Bátai^1,2^, Laura Kiss^1^, Luca Varga^1^, Ákos Nagy^1^, Jacob Househam^3^, Ann-Marie Baker^3^, Tamás László^1^, Anna Udvari^1^, Róbert Horváth^1^, Tibor Nagy^1,4^, Judit Csomor^5^, József Szakonyi^6^, Tamás Schneider^7^, Trevor Graham^3^, Donát Alpár^1^, Jude Fitzgibbon^8^, Ágota Szepesi^5*^ and Csaba Bödör^1*^

1 HCEMM-SU Molecular Oncohematology Research Group, Institute of Pathology and Experimental Cancer Research, Semmelweis University, Budapest, Hungary; 2 Department of Internal Medicine and Hematology, Semmelweis University, Budapest, Hungary; 3 Genomics and Evolutionary Dynamics Team, Centre for Evolution and Cancer, The Institute for Cancer Research, London, United Kingdom; 4 Department of Biochemistry and Molecular Biology, Faculty of Medicine, University of Debrecen, Debrecen, Hungary; 5 Institute of Pathology and Experimental Cancer Research, Semmelweis University, Budapest, Hungary; 6 Department of Dermatology, Venereology and Dermatooncology, Semmelweis University, Budapest, Hungary; 7 Department of Hematology and Lymphoma, National Institute of Oncology, Budapest, Hungary; 8 Barts Cancer Institute, Queen Mary University of London, London, United Kingdom; *these authors contributed equally

*Supplementary tables – available as one .xlsx document containing tables on different sheets*

Supplementary table 1. Shallow whole genome sequencing metrics and genome wide copy number burden results of primary cutaneous follicle center lymphoma samples (.xlsx).

Supplementary table 2. Copy number profile of primary cutaneous follicle center lymphoma (PCFCL, n=28) samples at cytoband resolution (.xlsx).

Supplementary table 3. Frequency, differential distribution and prognostic significance of copy number alterations in primary cutaneous follicle center lymphoma (PCFCL) and nodal follicular lymphoma (nFL) including the first sample of every patient (.xlsx).

Supplementary table 4. Frequency and differential distribution of copy number alterations in primary cutaneous follicle center lymphoma (PCFCL) and nodal follicular lymphoma (nFL) including all samples (.xlsx).

Supplementary table 5. Detailed immunophenotype of tumor samples from case #20 (.xlsx).

*Supplementary figures – available as one .pdf document containing all figures with legends*

Supplementary figure 1. Frequency histogram of copy number alterations in primary cutaneous follicle center lymphoma (.pdf).

Supplementary figure 2. Frequency of copy number alterations in primary cutaneous follicle center lymphoma differentiating focal (only observed as cytoband level change) and gross (observed at the chromosome arm level) alterations (.pdf).

Supplementary figure 3. Comparison of copy number burden metrics between nodal follicular lymphoma (nFL, n=64) and primary cutaneous follicle center lymphoma (PCFCL, n=28) samples (.pdf).

Supplementary figure 4. Comparison of copy number burden metrics between primary and recurrence primary cutaneous follicle center lymphoma (PCFCL) samples (.pdf).

Supplementary figure 5. Comparison of copy number burden metrics between cutaneous follicle center lymphoma (PCFCL) patients showing a localized disease course or distant spreading (.pdf).

Supplementary figure 6. Histopathological characteristics of patient samples in two representative cases with multiple tumor samples available for analysis during the disease course (.pdf).
